# Digital Technologies and Open Data Sources in Marine Biotoxins’ Risk Analysis: The Case of Ciguatera Fish Poisoning

**DOI:** 10.3390/toxins13100692

**Published:** 2021-09-30

**Authors:** Panagiota Katikou

**Affiliations:** Ministry of Rural Development and Food, Directorate General of Rural Development, Directorate of Research, Innovation and Education, Hapsa & Karatasou 1, 54626 Thessaloniki, Greece; pkatikou@otenet.gr

**Keywords:** Ciguatera Fish Poisoning, digital technologies, open data, risk analysis, marine biotoxins

## Abstract

Currently, digital technologies influence information dissemination in all business sectors, with great emphasis put on exploitation strategies. Public administrations often use information systems and establish open data repositories, primarily supporting their operation but also serving as data providers, facilitating decision-making. As such, risk analysis in the public health sector, including food safety authorities, often relies on digital technologies and open data sources. Global food safety challenges include marine biotoxins (MBs), being contaminants whose mitigation largely depends on risk analysis. Ciguatera Fish Poisoning (CFP), in particular, is a MB-related seafood intoxication attributed to the consumption of fish species that are prone to accumulate ciguatoxins. Historically, CFP occurred endemically in tropical/subtropical areas, but has gradually emerged in temperate regions, including European waters, necessitating official policy adoption to manage the potential risks. Researchers and policy-makers highlight scientific data inadequacy, under-reporting of outbreaks and information source fragmentation as major obstacles in developing CFP mitigation strategies. Although digital technologies and open data sources provide exploitable scientific information for MB risk analysis, their utilization in counteracting CFP-related hazards has not been addressed to date. This work thus attempts to answer the question, “What is the current extent of digital technologies’ and open data sources’ utilization within risk analysis tasks in the MBs field, particularly on CFP?”, by conducting a systematic literature review of the available scientific and grey literature. Results indicate that the use of digital technologies and open data sources in CFP is not negligible. However, certain gaps are identified regarding discrepancies in terminology, source fragmentation and a redundancy and downplay of social media utilization, in turn constituting a future research agenda for this under-researched topic.

## 1. Introduction

The rapid acceleration of digital technologies, evidenced more intensely during the past decade, globally permeates every private and public organization, transforming their daily working practices, at the same time reshaping social interactions and citizens’ expectations [1,2]. Digital tools, among which the Internet, social media, mobile computing, big data, data analytics, and numerous others, open up a fascinating world of innovation opportunities with a significant impact on multiple aspects of contemporary societies [1,2,3]. This overwhelming penetration of information and communication technologies (ICTs) in everyday life is altering the information sharing preconditions and can technically support more collaborative cultures of information production and dissemination, thus shifting the focus from technology itself to strategies for its exploitation [4].

Substantial implementation of digital technologies in government/public sector operations, commonly intersecting with the e-Government concept, entails the use of public information systems and the creation of open data repositories, to serve as tools supporting the fundamental principles of transparency, participation and collaboration [4,5]. Efficient incorporation and interoperability of these tools can improve decision-making, by providing policy-formulators with ample data to address complex problems and to design effective public policies in various governmental disciplines [5,6]. The public health sector, and particularly food safety authorities, should be no exception. Digital technologies and open/big data can be of utmost importance in risk analysis processes, the latter considered necessary to proactively refine and optimize food safety and legislation, rather than maintaining a reactive management approach [7,8].

Emergence of new pathogenic microorganisms and the unintentional presence of chemical contaminants constitute major biological and chemical hazards, respectively, significantly challenging global food safety. Natural toxins, and marine biotoxins (MBs) in particular, comprise a distinct type of food hazard, in the sense that they are chemical toxic substances but are of biological origin [8]. MBs are synthesized by specific marine microorganisms, mainly microalgae (usually termed as phytoplankton) and a few bacterial species. Under certain favorable environmental conditions, toxic or harmful algae may proliferate and aggregate to form dense cell assemblages, commonly known as ‘harmful algal blooms’ (HABs), accompanied by MBs production able to contaminate seafood, resulting in a serious health threat to consumers. MBs could cause severe human intoxications, such as amnesic, diarrheic, azaspiracid, neurotoxic and paralytic shellfish poisonings and Ciguatera Fish Poisoning (CFP) [9]. MBs are in general heat-stable compounds, resistant to common food processing technologies, whereas no antidote exists to reverse their effects in humans [10]. Illness prevention is thus essential to manage MB-related public health risks, with risk analysis being an irreplaceable tool in the arsenal of public authorities pursuing mitigation of HABs’ negative impacts [9,10].

Worldwide, CFP is the most prevalent biotoxin-related seafood poisoning, resulting from the consumption of seafood contaminated by its associated toxins, known as ciguatoxins (CTXs) [11]. Despite the significant under-reporting of cases due to a lack of diagnostic methods, CFP is estimated to annually affect approximately 50,000–500,000 people [12]. Historically, this syndrome is mainly encountered in tropical and subtropical areas. In the recent past, however, a geographical expansion of CFP in more temperate areas has been evidenced. Factors such as climate change, but also some anthropogenic activities, are incriminated for altering the geographical distribution of the causative organisms, which are dinoflagellates of the genera *Gambierdiscus* and *Fukuyoa*, as well as the migration patterns of ciguateric fish [13,14]. Additional factors influencing the occurrence of CFP in non-endemic areas are related to international trade and consumption of imported ciguateric fish species in non-tropical areas and/or travelers returning from CFP endemic areas [11,12]. Current reports of CFP in temperate waters of the Canary Islands (Spain) and the Madeira archipelago (Portugal), accompanied by the documented presence of *Gambierdiscus* and *Fukuyoa* spp. in the Mediterranean Sea, constitute CFP being emerging hazard in European waters, thus necessitating the adoption of official policies to manage the potential risks [12,15]. Nonetheless, the European Food Safety Authority (EFSA) highlighted scientific data inadequacy among the reasons hindering the development of appropriate human health protection strategies against CFP [16], whereas the problems of CFP cases’ under-reporting and information sources’ fragmentation are emphasized by both researchers and policy-makers [12,16]. Taking into account these shortcomings, in combination with the significant health, socioeconomic and socio-cultural impacts of CFP, as well as its increasing emergence in non-endemic areas, improvements in data collection and availability are evidently required at a global level, to allow for more efficient risk monitoring and mitigation [11,12]. In this context, recognizing some of the technological developments able to generate CFP data and assist their dissemination, as well as compiling a roster of openly accessible information sources for CFP, could facilitate the efforts to tackle the weaknesses identified.

Doubtlessly, developments in digital technologies and open data sources amplified the volume of potentially exploitable scientific information for MBs risk analysis purposes. However, bibliographical references on the advancements achieved with the participation of such means in counteracting CFP-related hazards are scattered, whereas, to date, no substantial summary or research study has cumulatively investigated their utilization. The problem addressed in the present work thus relates to examining digital technologies’ and open data sources’ utilization in CFP research associated with risk analysis tasks. The research question answered in this review, therefore, is “What is the current extent of digital technologies’ and open data sources’ utilization within risk analysis tasks in the MBs field, particularly on CFP?”

The absence of targeted review articles and scarcity of structured information on the topic necessitated an in-depth literature investigation, within both peer-reviewed publications and grey literature documents to accomplish this study. For the purposes of this review, grey literature is defined according to the Prague definition, “manifold document types produced on all levels of government, academics, business and industry in print and electronic formats that are protected by intellectual property rights, of sufficient quality to be collected and preserved by libraries and institutional repositories, but not controlled by commercial publishers; i.e., where publishing is not the primary activity of the producing body” [17] (p.11), typically including “conference abstracts, presentations, proceedings; regulatory data; unpublished trial data; government publications; reports (such as white papers, working papers, internal documentation); dissertations/theses; patents; and policies & procedures” [18] (para.2).

The structure of the review is as follows: the next section provides brief background knowledge on the concepts of digital technologies, open data sources and risk analysis, viewed from a public health and food safety perspective, to assist in determining the appropriate keywords for the literature investigation. The subsequent two sections describe the research methodology employed and present the bibliographical analysis results. Finally, the findings are discussed, and relevant future research is suggested.

## 2. Background

### 2.1. Digital Technologies

Digital technologies are broadly defined as “combinations of information, computing, communication, and connectivity technologies” [19]. An initial review of recent research mainly focusing on the public health and food safety contexts, but also at wider level, reveals that concepts such as ‘technology’, ‘digital technologies’, ‘ICT’, ‘information technologies’, ‘digital media’ and ‘digital tools’ are used interchangeably to refer to a broad set of digital devices and applications, such as websites, databases, blogs, online platforms, mobile/wearable devices, mobile phones, social media and the Internet [20,21,22,23,24]. Digital technologies/ICTs are also strongly intertwined with the ‘digital transformation’ and ‘digitalization’ concepts. Indeed, ‘digital transformation’ is defined as the use of digital technologies/ICTs to enable changes and improvements for achieving business and/or organizational goals [25] and ‘digitalization’ as the sociotechnical process of using digital infrastructures [1]. In this context, the terms ‘digital transformation’ and ‘digitalization’ may also constitute relevant keywords for investigating digital technologies utilization, as they are linkable to improvements and changes in work processes of organizations responsible for risk analyses. Digital technologies’ proliferation enhances the quality and quantity of daily generated data, creating conditions of information abundance, able to significantly facilitate public authorities’ decision- and policy-making processes [19,26].

### 2.2. Open Data Sources

Open data refer to “non-privacy-restricted and non-confidential data produced with public money by public and/or private organizations and made available without any usage or distribution restrictions” [6] (p. 258). Open data, frequently termed also as ‘public data’, can be enriched with data from other sources, resulting in the emergence of large datasets, known as ‘big data’ [7]. The latter present specific needs for processing, curation, linking, visualization and maintenance, as their sizes overpass common software tools’ abilities, whereas value is generated by the combination of different datasets [19,26].

Public policy development frequently relies on ‘open data’ and ‘big data’ availability, being indispensable tools for public organizations. Ample open data in diverse formats are stored in repositories on national or international organizations’ websites and also can be exploited by other public institutions, thus counteracting unnecessary duplication and associated costs [6]. However, food safety data and information are generally scattered across the food, health and agriculture sectors, with limited interoperability. Consequently, public authorities in charge of food safety-related risk analysis tasks ordinarily resort to multiple open access scientific resources, such as research project websites, online databases, open-access journals, dissertations or other published material, to obtain up-to-date technical information. Efficient access to such sources is granted by the growth of digital technologies [27,28]. For the purposes of the present review, the ‘open data sources’ concept will also extend to ‘big data’, including those forms of ‘open-source’ and ‘open-access’ scientific data and software freely available in the public domain [7]. Consequently, the search for appropriate information based on the keywords selected, will also encompass results of common Internet search engines, besides the literature databases [29].

### 2.3. Risk Analysis in Food Safety

Risk analysis is a powerful science-based tool for reaching sound, consistent solutions to food safety problems. The Codex Alimentarius Commission defines risk analysis in a food safety context as “a process consisting of three components: risk assessment, risk management, and risk communication” [30] (p. 120). More precisely, risk analysis in food safety is a systematic, disciplined decision-making approach, used to estimate human health and safety risks, to identify and implement appropriate measures for risk control, and to communicate with stakeholders about the risks and measures applied [31]. ‘Risk assessment’ is the science-based component of risk analysis, comprising hazard identification and characterization, exposure assessment and risk characterization. ‘Risk management’, on the other hand, involves weighing policy alternatives in consultation with relevant stakeholders, according to the risk assessment outcomes and other factors relevant for consumers’ health protection, towards selecting appropriate prevention and control options. Lastly, ‘risk communication’ entails an “interactive exchange of information and opinions throughout the risk analysis process concerning risk, risk-related factors and risk perceptions, among risk assessors, risk managers, consumers, industry, the academic community and other interested parties, including the explanation of risk assessment findings and the basis of risk management decisions” [30,31].

Food safety risk analyses are carried out by national, regional and international authorities, depending on the nature and localization of the specific risk examined [31]. Scientific knowledge on the food issue identified is considered a prerequisite for successful risk analysis; therefore, aggregation of the largest possible appropriate datasets is essential [28]. Strategies to obtain data on food contaminant issues, particularly MBs, require multidisciplinary approaches combining scientific information from fields such as environmental sciences, biology, chemistry, veterinary science, public administration, epidemiology, public health and toxicology. Data collection can present significant difficulties due to frequent gaps identified in information availability; in this context, the exploitation of digital technologies and open/big data sources may catalyze these efforts [10,28,31].

## 3. Literature Research Method

The current state of digital technologies and open data utilization in the field of CFP risk analysis was envisaged by a systematic literature review conducted according to previously established principles [29,32]. Three main steps were followed: (i) selecting appropriate keywords and combinations thereof; (ii) choosing source database(s) and running the searches; and (iii) analyzing the results.

The literature review protocol employed is detailed in Table 1. The focus period was set from 2010 to date (mid 2021). The main keywords identified within the background section were divided into five groups, namely, “Digital technologies”, “Open data”, “Risk analysis”, “Biotoxins” and “Ciguatera”, according to the concepts comprising the research topic. Each of the keywords from Groups 1–3 was combined with one or more keywords from the remaining two groups to retrieve the articles of interest, utilizing the Boolean operators “AND” and “OR” (on a case basis) to produce more focused results. Searches were performed separately for each combination of keywords and applied to the journals’ abstracts, title and keywords, using the Scopus abstract and citation database of peer-reviewed literature. All subject areas were selected, due to the multidisciplinary character of this research.

**Figure 1:**
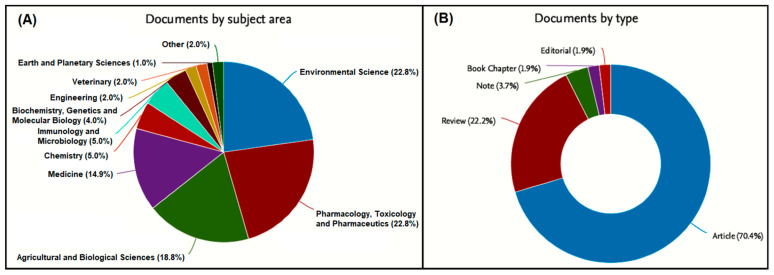
Combined search for the “Risk analysis”. “Biotoxins” and “Ciguatera” group keywords by (**A**) subject areas and (**B**) types of retrieved documents. “Other” indicates research disciplines not individually mentioned (refer to Table 1 for details).

This strategy yielded only one result when the keywords of the “Digital technologies” or “Open data” groups were combined with keywords of the “Biotoxins” or “Ciguatera” groups. A much higher total number of articles was obtained, as expected, when the “Risk analysis” group keywords were looked-up in combination to those of the “Biotoxins” and “Ciguatera” groups. Searches were merged, and after removing the duplicates, 88 articles of multiple types and subject areas remained (Figure 1). The articles’ abstracts were carefully read to assess their relevance and to exclude articles containing the selected keywords in another semantic way, shortening down the list to 28 articles. After full-text examination for the presence of appropriate information, more were excluded as “out of topic”, with only 11 studies remaining, a rather expectable outcome considering the narrowness of the field and the specialized nature of the research topic. An additional search in the “Pubmed” database, using the same keyword combinations, yielded five further articles. Thereafter, a thorough Google search was conducted, combining in pairs all the above keywords and some additional terms (e.g., database, smartphone, website, satellite imaging, machine learning), to obtain further material from both the scientific and grey literature, such as press releases, health and fishery authorities’ websites, local media, project documents, codes of practice, etc. Finally, reference lists of all selected documents were reviewed to find other articles of interest, whereas their citations in later publications were also evaluated for inclusion in this review [32].

Articles considered relevant contained at least one reference to data input for CFP risk analysis or its individual components (assessment, management, communication) obtained by means of digital technologies and/or open data sources, such as websites, databases, software, social media, specific pieces of digital equipment, etc. It is noted that this research only considers digital equipment utilization in terms of mass-market tools, such as computers and portable digital devices (e.g., notebooks, smartphones, tablets); the use of sophisticated analytical equipment, such as liquid chromatographs and mass spectrometers, although largely incorporating digital components (computerized appliances, support PCs and processing software), is beyond the scope of this work. Similarly, statistical analysis software packages, as well as common office-computer software for word processing, spreadsheets creation, etc., are not included in this literature review, as their use is a prerequisite in CFP data generation. In this context, the above strategy resulted in a final list of 38 articles, of which only 19 were openly accessible to the regular public. In the next step, information of relevance was abstracted from the selected documents and contents were analyzed within the identified research concepts’ framework, as presented in the following section.

## 4. Result

Keywords found in the 38 articles meeting the eligibility criteria are summarized in Table 2. Notably, references connected to digital technologies were fewer than those categorized within the open data sources concept, with 16 and 33 articles, respectively, whereas 11 articles contained keywords of both groups. ‘Database’ was the keyword most encountered, with 28 articles, while the highest incidence keyword combination was ‘website’–‘database’, with five articles. Further details are provided in Supplementary Materials Table S1.

### 4.1. Digital Technologies

Only three results [33,34,35] were finally obtained using the exact keywords indicated within the digital technologies concept, combined with those related to ciguatera and risk analysis (Table 1); on the other hand, the extended search for specific digital tools retrieved 13 studies containing the terms ‘software’, ‘smartphone’ and ‘website’, as semantic content relevant to the production, processing and/or communication of the data necessary for CFP risk analysis (Table 2). Interestingly, only two articles combining ‘social media’ and ‘ciguatera’ within the context of risk analysis were retrieved, despite the existence of several CFP-relevant Facebook and Twitter accounts (Supplementary Materials Table S2) and the popularity of social media [34,35]. The first one referred to social media mechanisms for food/waterborne complaints surveillance and indicated specific social media accounts serving this purpose [34]. The second one only mentioned the appearance of anecdotal reports of CFP cases on social media, such as online fishing for a, where fishers comment on their own experiences providing the opportunity for broader data collection and risk communication, but without pointing to any specific social media accounts [35].

The term ‘software’ in risk analysis-related CFP studies primarily concerned programs used for molecular/phylogenetic identification of ciguateric fish and CTX-producing microalgae and secondly web applications assisting record-keeping and communication regarding the presence of ciguateric fishes in trade operations [36,37,38]. Accurate identification of high-risk fish species implicated in CFP and the ability to prevent these from reaching the market, according to regional legislative requirements, are critical in CFP risk assessment, management and communication; therefore, software-based tools can facilitate risk analysis processes [39,40].

Generally, instances of ‘website’ in the selected articles referred to governmental and organizations’ internet pages containing diverse scientific information, including CFP case reports, epidemiological and environmental data, outbreaks occurrence and advice to consumers, as well as other public health data, all being major inputs to CFP risk analysis components [11,39,41,42,43,44,45]. Nevertheless, ‘website’ was also used by some authors to denote any type of online-available content, such as public databases or even open data portals (Table 3) [41,44,45]. Furthermore, although fishing bans related to geographical origin (known toxic locations), high-risk fish species and fish size restrictions constitute fundamental measures in terms of CFP risk management in endemic areas [11], often communicated to relevant stakeholders through designated websites, social media or applications belonging to public agencies, no relevant articles were retrieved in the literature (scientific or grey) referring to these specific risk communication actions.

Widely marketed digital tools, such as smartphones, have recently emerged as attractive analytical platforms, which in the future may revolutionize food safety control by enabling citizens without any expertise to perform screening tests [46]. A number of smartphone-based devices or assays have already been developed for various contaminants, including marine toxins [28,46,47] and CTXs, in particular [48]. It should be noted that, currently, smartphones cannot be used on their own to detect food contaminants, without the contribution of some auxiliary part or hand-held device, such as portable electrochemical or optical sensors [28,47,48,49]. However, they possess independent power sources, computing power, flash-light cameras (i.e., optical systems with constant light sources), web access and wireless data communication, being powerful alternative analytical tools, able to radically change food testing. Although smartphone apps for CTXs testing are not yet commercially available, the future ability of consumers to screen fish for CFP is expected to improve food security and increase public awareness, facilitating also risk assessment and management [47,49].

### 4.2. Open Data Sources

Occurrence of keywords belonging to the ‘open data sources’ group combined to ‘ciguatera’ was extensively searched, but no studies were found containing ‘open data’, ‘public data’ and ‘open source’, whereas only one publication (a Master’s thesis) included the term ‘big data’ [50]. On the other hand, searching specifically for ‘database’, after exclusion of instances related to literature/journal databases, resulted in 28 publications containing at least one reference to a data source compliant to the ‘open data sources’ concept of the present work [11,14,36,37,40,41,43,44,45,51,52,53,54,55,56,57,58,59,60,61,62,63,64,65,66]. Another relevant term encountered in a semantic fitting the concept was ‘dataset’ [67,68], a term frequently used interchangeably to ‘database’ [69], while the more general term ‘data’ was the only one present in other works containing records of CFP incidents derived from public databases [39,70]. A cumulative presentation of the open data sources found in the selected studies is included in Table 3, along with the geographic coverage and an attempt to categorize source types in compliance with the concept description of the present work, using terms as ‘open data portal’, ‘open documents repository’, ‘public/open source software’, etc. This summary is provided in order to explicitly demonstrate the extent, diversity and fragmentation of the available sources, as well as the type of data available for risk analysis purposes, but also to facilitate future CFP research with regard to data retrieval. To our knowledge, all sources included in Table 3 are openly accessible to the regular public, although in some cases a user registration may be required.

The variety of open data source types found in the studied literature (Table 4) indicates that the data derived thereof are sufficiently exploited in the field of CFP research and risk analysis. Evidently though, the terms ‘open data’ ‘public data’, ‘open source’ and ‘big data’, commonly used in relevant social sciences’ research, are practically unknown to authors involved in this field. On the other hand, ‘database’ was the most frequently used term to describe such information sources, with some articles specifically referring to databases as ‘public’ [33,55,56], ‘web-based’ [34], ‘online’ [59], ‘internet’ [60], ‘electronic’ [61] or ‘open access’ [65], whereas ‘online data’ was also used in one case [43].

Geographical coverage of the open data sources found in the selected articles ranged from worldwide to regional, with the majority of non-global coverage sources focusing their data on areas located in the American and Oceania continents, where CFP is long encountered and considered endemic. In contrast, sources targeting for instance European countries, where CFP issue has recently emerged, are scarcer.

The nature of the CFP-related data contained within the identified open data sources varied widely, including data on taxonomy and identification of marine species (fish and microalgae) [11,14,33,36,37,39,43,52,55,56,57,59,60,61,66], epidemiology and outbreaks occurrence [11,14,33,34,35,39,40,41,43,44,51,52,53,54,55,58,60,61,63,64,65,70], HAB events [11,14,43,53,60,62,63,64,65,66], climate and environment (temperature, salinity, water quality monitoring, benthic habitats) [33,41,45,50,51,58,62,67,68], public policies and risk mitigation strategies [11,33,43,61] as well as general information on CFP’s public health perspective to aid risk communication to the public [11,34,39,41,43,44,51,61,70].

Plurality in open data sources of a similar nature containing data on different regions is also noteworthy, indicating that efforts to collect data, especially those related to CFP surveillance, epidemiology, case reports and outbreaks incidence, are localized and fragmented, even within the same country, such as the data sources of different states within the USA. Conversely, the evident absence of instances of open data sources in certain CFP-susceptible areas of the world, such as some African and Asian countries of the West Indian Ocean, is also notable. Significant redundancies are also encountered, primarily with regard to climate data, and sea surface temperatures in particular, where at least five different sources are available at a worldwide level. Similarly, at least four different open sources exist for fish or algal species taxonomy and identification. As such, policy-makers and researchers undertaking international risk analysis tasks are commonly obliged to resort to multiple information sources and spend considerable time to obtain the required amount of data. On the other hand, discrepancies may also occur between data from different sources, the resolution of which may create an additional burden in order to obtain acceptable data quality for risk analysis purposes.

## 5. Discussion

### 5.1. Research Question Revisited

This review addressed the research question, “What is the current extent of digital technologies’ and open data sources’ utilization within risk analysis tasks in the MBs field, particularly on CFP?” Although the commonly expected terminology was almost absent in the relevant bibliography, modifying the search keywords revealed the existence of several CFP risk analysis-related publications, 38 in total, where the data input originated from the use of diverse digital tools and sources. As such, it appears that the current utilization of digital technologies and open data sources in the investigated field is generally not negligible, which reasonably answers the research question.

### 5.2. Further Remarks on the Findings

The aforementioned findings demonstrate that exploitation of digital technologies and open data sources in CFP risk analysis and policy-making studies is not negligible, with their utilization being more widespread in scientific works targeting CFP-endemic areas [40,42,44,45,50,51,52,60,61,64,66,67,68,70]. Nevertheless, the pronounced shortage of published works on CFP referring to common social sciences terminology, such as ‘digital technologies’, ‘digital transformation’, ‘open data’ and ‘big data’, in conjunction with the use of the general terms, e.g., ‘website’, ‘database’ and ‘dataset’, is indicative of an unfamiliarity with these terms regarding the scientific community creating/uploading information and datasets of interest on the Internet, as well as researchers utilizing the data obtained by these sources. Lack of uniformity between the social and natural sciences’ terminology is not a new issue; in fact, it forms part of a long-observed general gap between social and natural sciences, thus highlighting the necessity to adopt more transdisciplinary and collaborative approaches across research fields belonging to environmental/marine sciences, toxicology, public health and social sciences [62,71,72].

The fragmented dispersion of data related to CFP surveillance, epidemiology and outbreaks occurrence encountered in the open data sources identified in this literature review, has also been suggested in previous studies. In fact, under-reporting or inconsistent and fragmented recording of CFP cases has been attributed to the absence of formal epidemiological and surveillance methods and a lack of clinical protocols and experience, whereas the need to establish an international register for CFP intoxication cases and also consolidate monitoring of HAB events at a global level is largely emphasized [11,43,53,55,62,73].

Surprisingly only two instances of ‘social media’ related to CFP [34,35] were found within both peer-reviewed and grey literature publications, suggesting that these digital tools could be underexploited in CFP risk analysis. In fact, food safety agencies already use social media, such as Facebook and Twitter, for risk communication with the general public on food safety issues [26,74], and CFP is no exception. Several CFP-relevant accounts already exist in social media (Table S2), and CFP risk communications, such as notifications of fishing bans or advice to fishers on species and areas at risk, are not uncommon, especially in CFP-endemic regions. On the other hand, online reporting of CFP cases in social media accounts [34], as well as exchange of CFP-related experiences through posts on fishing forums, are also frequent. Evidently, this does not seem to be adequately reflected in the literature, indicating that the impact of social media in the CFP field may constitute a scientific knowledge gap, requiring further research to elucidate their dynamics as data-providing sources and communication tools in CFP policy-making.

## 6. Limitations, Conclusions and Future Research

To our knowledge, to date, no previous works have summarized the utilization of both digital technologies and open data sources in tasks relevant to risk analysis, regarding either MBs or specifically CFP. As such, this review constitutes an initial attempt towards documenting the utilization extent of these tools in CFP risk analysis, according to the currently available literature, but certainly cannot be considered an exhaustive summary of their contribution or an assessment of their effectiveness in this HAB management field. We anticipate this first theoretical approach to trigger further investigation, entailing empirical data, in order to provide concrete evidence on the extent of the interactions between developments in the digital world and their practical applications in the diverse natural sciences fields, including MBs and CFP in particular. In this context, a structured research strategy is required to thoroughly evaluate the impact level of such ICT tools in a qualitative and quantitative way. To achieve this objective, the following approaches are suggested:

(1) Interviewing relevant stakeholders, such as experts, public administrators and researchers, involved in the field of CFP risk management, in order to assess (a) their degree of familiarization with the terminology related to digital technologies and open data sources; and (b) their understanding, own use and perception of specific digital technologies and open data sources. This assessment can be accomplished by means of structured questionnaires, containing both multiple-choice/close-ended (with a rating scale) and open-ended questions, as well as free statements, subsequently followed by content and statistical analysis of the responses obtained. Participation could also be expanded using online forms and/or email-invited questionnaires to more effectively target expert audiences.

(2) Introducing qualitative and quantitative criteria to create a framework for evaluating the impact of the given digital technologies and open data sources and subsequent application of this model to analyze the answers obtained within the context of the available literature.

Unequivocally, capitalization of technological progress is the way forward to scientific progress in the modern world. On this basis, accessibility to and exploitation of digital tools and open/big data are synergistically expected to derive innovative applications and services, aiming to facilitate risk analysis and policy-making procedures in the field of food safety, similarly to the progress envisaged in the fisheries sector by the implementation of emerging data technologies, such as blockchain, data mining and artificial intelligence [75]. In the framework of the gaps identified within the present study, research towards consolidation of the currently fragmentary open data sources, such as epidemiological and HAB presence databases, at a worldwide level, can support more robust practices towards mitigation of the CFP problem. On the other hand, embracing the social media potential to strengthen data collection and enhance risk communication channels in the CFP sector is also considered crucial, and definitely requires further scientific research in order to both capture the benefits and tackle the challenges involved. Finally, and most importantly, transdisciplinary collaboration is essential to bridge the evident chasm between humanities and natural sciences, with establishing mutually accepted terminology and definitions for concepts of common interest as a starting point.

## Figures and Tables

**Table 1:** Literature review protocol (Scopus search).

Item	Description				
Time Period	2010 to Date				
Boolean Operators	AND/OR				
Keywords	1: Digital Technology	2: Open Data	3: Risk Analysis	4: Biotoxins	5: CFP
	Digital technolog* ^1^	Open data	Risk analysis	Biotoxin*	Ciguatera
	Digital tool*	Big data	Risk management	Marine toxin*	Ciguatoxin*
	Digital media	Public data	Risk communication	Phycotoxin*	
	Digital transformation	Open source*	Risk*		
	Digitalization	Data*			
	Information technolog*				
	ICT(s)				
	Social media				
Language	English				
Availability	Documents available online as full text				
Research Discipline	All subject areas (indicatively: Pharmacology, Toxicology and Pharmaceutics; Agricultural and Biological Sciences; Environmental Science; Medicine; Chemistry; Immunology and Microbiology; Biochemistry, Genetics and Molecular Biology; Engineering; Veterinary; Earth and Planetary Sciences; Chemical Engineering; Health Professions; Multidisciplinary; Computer Science; Arts and Humanities; Social Sciences)				
Exclusion Criteria	Articles unrelated to intended subject (keywords with other semantic way)				
Publication type	All available types (Article, Review, Book, Book chapter, Conference paper, Conference review, Letter, Editorial, Note, Short survey, Business article or Press, Erratum, Data paper)				

^1^ An asterisk was used as a wildcard symbol to retrieve all possible variations of the relevant search term.

**Table 2:** Keywords identified in articles meeting the eligibility criteria.

Keyword Group/Keywords Found	Number of Studies *
**Digital Technologies**	**16**
Software	3
Smartphone	3
Website	7
Digital technologies	1
Social media	2
**Open Data**	**33**
Database	28
Big data	1
Dataset/data	4
**Total Selected**	**38**

* Number of studies containing keywords from individual groups cumulatively exceed the total number of selected articles, as some studies contain keywords of both groups.

**Table 3:** Open data sources used in CFP literature related to risk analysis (sources referred to as ‘websites’ are included).

Geographic Coverage	Organization/Website ID	Source Type	Nature of CFP-Related Data	Link ^(1)^	Reference ^(2)^
Worldwide	3i Interactive Key and Taxonomic Database Software	Public database and software	Taxonomic species identification	http://dmitriev.speciesfile.org/ http://dmitriev.speciesfile.org/key.asp?key=Bacillariales&lng=En&i=1&keyN=1	[37] and PW
Worldwide	AlgaeBase	Public database	Algal species information	https://www.algaebase.org/ http://www.algaebase.org/search/genus/detail/?genus_id=45535	[33]
Worldwide	Barcode of Life Data System (BOLD)	Public database and open data portal	DNA-based species identification	http://www.boldsystems.org/	[36,56,57]
Worldwide	Bayesian Evolutionary Analysis Sampling Trees (BEAST)—Tracer	Open-source software	Molecular sequencing/Phylogenetic analysis	http://beast.community/index.html http://beast.community/tracer	[36]
Worldwide	Bayesian Tip-association Significance testing (BaTS)	Open-source software	Phylogenetic analysis	http://evolve.zoo.ox.ac.uk/Evolve/BaTS.html	[36] and PW
Worldwide	FishBase—A Global Information System on Fishes	Public database	Ciguateric fish species	https://www.fishbase.in/search.php (Information by topic/Uses/Ciguatera) https://www.fishbase.in/Topic/List.php?group=27	[36,39,55,57,61]
Worldwide	FishSource—Sustainable Fisheries Partnership	Public database	Status of fisheries, fish stocks, and aquaculture	https://www.fishsource.org/	[70]
Worldwide	GenBank	Public database and open data portal	Genetic sequence (algal species and ciguateric fish)	https://www.ncbi.nlm.nih.gov/genbank/ https://www.ncbi.nlm.nih.gov/genbank/ftp/	[56,57,59] and PW
Worldwide	Global Biodiversity Information Facility (GBIF)—Integrated Publishing Toolkit (IPT)	Open data portal	Biodiversity (reef fish, invertebrates and algae, HABs, water quality)	https://www.gbif.org/ https://www1.usgs.gov/obis-usa/ipt/ https://www.gbif.org/dataset/search	[41,50] and PW
Worldwide	Intergovernmental Oceanographic Commission of UNESCO—Harmful Algal Bloom Programme/IOC-UNESCO Taxonomic Reference List of Harmful Micro Algae	Public database	Harmful microalgal species information	http://www.marinespecies.org/hab/	[66]
Worldwide	MrBayes: Bayesian Inference of Phylogeny	Open-source software	Phylogenetic and evolutionary models	http://nbisweden.github.io/MrBayes/index.html http://nbisweden.github.io/MrBayes/download.html	[36] and PW
Worldwide	National Aeronautics and Space Administration (NASA)/Ocean Color & Physical Oceanography Distributed Active Archive Center (PO.DAAC)	Public database, maps and open data portal	Sea surface temperature, salinity, density	https://oceancolor.gsfc.nasa.gov/ https://oceandata.sci.gsfc.nasa.gov/ https://podaac.jpl.nasa.gov/	[45]
Worldwide	National Aeronautics and Space Administration (NASA)/Worldview EOSDIS (Worldview app) and Earth Science Data Systems (ESDS) program (Earthdata)	Public database, maps and open data portal	Satellite-derived sea surface temperature	https://worldview.earthdata.nasa.gov/ https://search.earthdata.nasa.gov/search	[33] and PW
Worldwide	National Oceanic and Atmospheric Administration (NOAA)—Coral Reef Watch (CRW)	Public database and maps	Coral Reef Satellite Monitoring	https://coralreefwatch.noaa.gov/satellite/index.php	[67]
Worldwide	NOAA—Physical Sciences Laboratory (PSL)	Open data portal	Climate, sea surface temperature	https://psl.noaa.gov/data/gridded/ https://psl.noaa.gov/data/gridded/data.noaa.oisst.v2.html	[51,67]
Worldwide	NOAA—National Centers for Environmental Information (NCEI) (formerly National Climate Data Center)	Public database, maps and open data portal	Environmental (atmospheric, coastal, geophysical & oceanic)	https://www.ncei.noaa.gov/ https://www.ncei.noaa.gov/access https://www.ncei.noaa.gov/products/world-ocean-database	[45,68] and PW
Worldwide	Ocean Biodiversity Information System (OBIS)	Open data portal and public maps	Toxic algal species occurrence	https://obis.org/ https://mapper.obis.org/ https://obis.org/manual/access/	[14,60,66]
Worldwide	The Met Office UK—Hadley Centre Sea Ice and Sea Surface Temperature data set (HadISST)	Open data portal	Climate, sea surface temperature	https://www.metoffice.gov.uk/hadobs/hadisst/ https://www.metoffice.gov.uk/hadobs/hadisst/data/download.html	[58] and PW
Worldwide	UNESCO–IOC–ICES–PICES/Harmful Algae Event Database (HAEDAT)	Public database and maps	HAB events	http://haedat.iode.org/ http://envlit.ifremer.fr/var/envlit/storage/documents/parammaps/haedat/	[11,14,53,60,63,65,66]
Worldwide	World Register of Marine Species (WoRMS)	Public database	Marine organisms’ taxonomy (algae and fish)	http://www.marinespecies.org/index.php	[52]
European Union	European Commission—Rapid Alert System for Food and Feed (RASFF)	Public database	Occurrence in foods	https://webgate.ec.europa.eu/rasff-window/portal/?event=SearchForm&cleanSearch=1 https://webgate.ec.europa.eu/rasff-window/portal/?event=SearchByKeyword&NewSearch=1&Keywords=cigua	[53,54] and PW
France, Italy, Monaco	RAMOGE/Regional workshop on monitoring and management strategies for benthic HABs	Open documents repository	Various informational	http://www.ramoge.org/fr/news.aspx?id=112	[11]
Canary Islands	Gobierno de Canarias	Open documents repository	CFP occurrence data, general information on CFP intoxication in Canary islands	https://www.gobiernodecanarias.org/agp/sgt/temas/estadistica/pesca/index.html https://www.gobiernodecanarias.org/agp/sgt/galerias/doc/estadisticas/pesca/Estadistica-ciguatera-2017_2018.ods https://www3.gobiernodecanarias.org/sanidad/scs/contenidoGenerico.jsp?idDocument=bb1799ed-b4c0-11de-ae50-15aa3b9230b7&idCarpeta=3ec36999-d4e1-11e2-8241-7543da9dbb8a	[70]
America	Pan American Health Organization/Institutional Repository for Information sharing	Open documents repository	Various informational	https://iris.paho.org/	[11,44] and PW
America (various territories)	NOAA—Environmental Sensitivity Index (ESI) and Geographical Information System (GIS) Mapping	Public database, maps	Benthic habitat data	https://response.restoration.noaa.gov/esi_download	[68]
United States of America	Centers for Disease Control and Prevention (CDC)—National Center for Environmental Health	Open documents repository	General information, Ciguateric fish species, HAB events, statistics	https://www.cdc.gov/nceh/ciguatera/default.htm https://www.cdc.gov/nceh/ciguatera/fish.htm https://www.cdc.gov/habs/index.html	[11,34,43] and PW
United States of America	Centers for Disease Control and Prevention (CDC)—Foodborne Disease Outbreak Surveillance System	Open documents repository	Foodborne outbreaks’ occurrence	https://www.cdc.gov/fdoss/annual-reports/index.html	
United States of America	CDC—National Outbreak Reporting System (NORS)	Public database, maps and statistics, open data portal	Outbreaks’ occurrence by etiology, year, state, primary mode, setting	https://wwwn.cdc.gov/norsdashboard/ (select Etiology → Ciguatoxin)	
United States of America	CDC—One Health Harmful Algal Bloom System (OHHABS)	Open documents repository	HABs-related human and animal illnesses & environmental data	https://www.cdc.gov/habs/ohhabs.html https://www.cdc.gov/habs/ohhabs_tables_and_figures.html	[62]
United States of America	Food and Drug Administration (FDA)	Open documents repository	Public policies, Ciguateric fish species, Fish and Fishery Products Hazards and Controls Guidance (CFP included)	www.fda.gov/Food/GuidanceRegulation/GuidanceDocumentsRegulatoryInformation/Seafood/ucm2018426.htm https://www.fda.gov/media/80748/download https://www.federalregister.gov/documents/2013/11/22/2013-27913/guidance-for-industry-on-purchasing-reef-fish-species-associated-with-the-hazard-of-ciguatera-fish https://www.fda.gov/media/80637/download	[33,43] and PW
Florida/US	Florida Complaint & Outbreak Reporting System (FL-CORS)	Public database	Online food and waterborne illness complaint forms	https://www.flcors.com/Home.aspx https://www.flcors.com/FWSupport	[34]
Florida/US	Coral Reef Evaluation and Monitoring Project (CREMP)	Open data portal	Coral reefs	https://www1.usgs.gov/obis-usa/ipt/resource?r=coralreefevaluationandmonitoringproject-1999	[50] and PW
Florida/US	Southeast Environmental Research Center (SERC)—Water Quality Monitoring Network	Open documents repository	Water quality monitoring	http://serc.fiu.edu/wqmnetwork/ http://serc.fiu.edu/wqmnetwork/Report%20Archive/report%20index.htm	[50] and PW
Florida/US	NOAA—National Hurricane Center (NHC)	Public database, open data portal	Tropical cyclones reporting (North Atlantic and eastern North Pacific basins)	https://www.nhc.noaa.gov/ https://www.ncei.noaa.gov/access/search/index	[68]
Hawaii	NOAA—Central Pacific Hurricane Center	Public database, open data portal	Tropical cyclones reporting (central Pacific)	https://www.nhc.noaa.gov/	[68]
Hawaii	State of Hawaii/Department of Health Disease Outbreak Control Division	Open documents repository	Case reports and statistics	https://health.hawaii.gov/docd/resources/reports/summary-of-reported-cases-of-notifiable-diseases/	[11]
Caribbean countries	Caribbean Epidemiology Center (CAREC)	Open documents repository	Epidemiology	https://iris.paho.org/handle/10665.2/2961	[14,44,60]
Caribbean countries	Caribbean Public Health Agency (CARPHA) CARPHA EvIDeNCe portal	Open documents repository	Public health data	https://carpha.org/Portals/0/Documents/CARPHA-State_of_Public_Health_Inaugural_Report_2013.pdf http://carphaevidenceportal.bvsalud.org/	[14,60] and PW
Caribbean countries	Caribbean Coastal Ocean Observing System (CARICOOS)—Part of Integrated Ocean Observing System (IOOS)	Open data portal, public database and maps	Climate, sea surface temperature, algae index, chlorophyll concentration	https://www.caricoos.org/ https://www.caricoos.org/data-download https://www.caricoos.org/ecosystem-and-water-quality https://www.caricoos.org/#!?detail=SelectOceanColor	[41,68] and PW
Caribbean + Gulf of Mexico	University of South Florida- College of Marine Science-Optical Oceanography Laboratory/Satellite-based Sargassum Watch System (SaWS)	Open data portal, public database and maps	*Sargassum* sp. seaweed presence/changes	https://optics.marine.usf.edu/projects/SaWS.html Interoperability with CARICOOS: https://www.caricoos.org/oceans/observation/modis_aqua/ECARIBE/afai	[41]
Mexico	Gobierno de México/Comisión Federal de Protección Contra Riesgos Sanitarios (COFEPRIS)	Open documents repository	HAB events	https://www.gob.mx/cofepris/acciones-y-programas/antecedentes-en-mexico-76707	[60] and PW
Australia	Australian Government Department of Health (AGDH)/Communicable Diseases Intelligence	Scientific journal (open access)	Epidemiology, surveillance, prevention & control	https://www1.health.gov.au/internet/main/publishing.nsf/Content/cdi-search	[11,35] and PW
Australia	AGDH—OzFoodNet	Open documents repository	Foodborne diseases’ surveillance	https://www1.health.gov.au/internet/main/publishing.nsf/Content/cdna-ozfoodnet-reports.htm	
Australia	Safefish—National Ciguatera Fish Poisoning Research Strategy	Open documents repository	Risk mitigation information	https://www.safefish.com.au/-/media/fish-safefish/documents/technical-reports/national-ciguatera-fish-poisoning-research-strategy-final.ashx	[11] and PW
Australia	Sydney Fish Market	Open documents repository	Seafood handling guidelines (incl. CFP)	https://www.sydneyfishmarket.com.au/Seafood-Trading/Quality/Food-Safety https://www.sydneyfishmarket.com.au/Portals/0/adam/Content/41UIctIuJECV0p4vxMVS4Q/ButtonLink/Seafood%20Handling%20Guidelines.pdf	[33]
Cook Islands	Cook Islands Ministry of Health/Te Marae Ora	Open documents repository	Cases statistics 2000–2016 (by year & month)	https://www.health.gov.ck/wp-content/uploads/2018/01/2016-National-Health-Information-Bulletin.pdf	[67] and PW
Fiji	Ministry of Health and Medical Services of Fiji	Open documents repository	Outbreak response guidelines	http://www.health.gov.fj/publications/ http://www.health.gov.fj/wp-content/uploads/2018/08/Fiji-Communicable-Disease-Surveillance-and-Outbreak-Response-Guidelines-2016-1.pdf	[11]
French Polynesia	Institut Louis Malardé—Ciguatera website	Open documents repository, public database and maps	General information, Ciguateric fish species, epidemiology mapping, surveillance and statistics, various documents	http://www.ciguatera.pf/ https://www.ciguatera.pf/images/poissons/CATALOGUE%20BR%20.pdf https://www.ciguatera.pf/index.php/fr/consultation-et-declaration https://www.ciguatera.pf/index.php/fr/la-ciguatera/surveillance-et-statistiques https://www.ciguatera.pf/index.php/fr/la-ciguatera/videos-et-medias	[11,33,39,41,51,61,64]
New Zealand	New Zealand Ministry of Health/Institute of Environmental Science and Research Ltd. (ESR)/Public Health Surveillance	Open documents repository	Outbreaks occurrence at annual and monthly basis	https://surv.esr.cri.nz/surveillance/annual_outbreak.php https://surv.esr.cri.nz/surveillance/monthly_surveillance.php	[52] and PW
South Pacific	Pacific Community/South Pacific Epidemiological and Health Information Services (SPEHIS)	Open documents repository	Epidemiology, health data (years: 1974–1996)	https://www.spc.int/DigitalLibrary/PHD/Collection/SPEHIS_SIESPS	[11,40,44,55,58]
Hong Kong	The Government of the Hong Kong special administrative region—Center for Food Safety	Open documents repository	General information, code of practice	https://www.cfs.gov.hk/english/multimedia/multimedia_pub/multimedia_pub_fsf_69_02.html https://www.cfs.gov.hk/english/whatsnew/whatsnew_fsf/whatsnew_fsf_poison_fish.html	[11,61]
Hong Kong	The Government of the Hong Kong special administrative region—Department of Health	Open documents repository	Annual case reports, press releases	https://www.search.gov.hk/result?tpl_id=stdsearch&ui_lang=en&ui_charset=utf-8&gp1=dh_home&gp0=dh_home&site=dh_home&web=this&query=ciguatera	[11]

^(1)^ All links accessed on 25 September 2021. ^(2)^ “PW” indicates absent/broken/obsolete links in referenced works retrieved/updated by the present work.

**Table 4:** Types and total instances of open data sources identified in the 33 selected articles (refer to Table 3 for details).

Open Data Source Type	Total Instances
Open documents repository	22
Public database	22
Open data portal	15
Public maps	12
Public/open source software	5
Open access journal	1

## Supplementary Materials

The following are available online at https://www.mdpi.com/article/10.3390/toxins13100692/s1, Table S1: Main keywords present in the selected articles related to the present review concepts, Table S2: Indicative social media accounts potentially relevant to CFP risk analysis.
