# Supplementary material for: Digital Technologies and Open Data Sources in Marine Biotoxins’ Risk Analysis: The Case of Ciguatera Fish Poisoning
Source: Toxins (Basel). 2021 Sep 30;13(10):692. doi: 10.3390/toxins13100692 (PMC8539326; doi:10.3390/toxins13100692)
Supplement: Supplementary file 1 [file toxins-13-00692-s001.zip › toxins-1327730-supplementary.pdf]

Supplementary Materials: Digital Technologies and Open Data Sources in Marine Biotoxins' Risk Analysis: The Case of Ciguatera Fish Poisoning

Panagiota Katikou

Table S1. Main keywords present in the selected articles related to the present review concepts.

Keywords Group	Digital Technologies	Open Data	Risk Analysis	Biotoxins	Ciguatera
**Reference**/ First Author & Year [ref. no] [1]					
Anderson 2019 [41]	X (website)	X (database)	X (Risk management)	X (Marine toxin*)[2]	X (Ciguatera)
Anderson 2021 [63]		X (database)	X (Risk assessment)	X (biotoxin*)	X (Ciguatera)
Bano 2020 [49]	X (smartphone)		X (Risk assessment)	X (Marine toxin*)	X (Ciguatera)
Boucaud-Maitre 2018 [64]		X (database)	X (Risk)	X (biotoxin*)	X (Ciguatera)
Bresnan 2021 [65]		X (database)	X (Risk assessment)	X (Marine toxin*)	X (Ciguatera)
Cabrera-Suárez 2019 [38]	X (software)		X (Risk analysis)	X (Marine toxin*)	X (Ciguatera)
Chan 2015 [42]	X (website)		X (Risk assessment)		X (Ciguatera)
Chinain 2021 [11]	X (website)	X (database)	X (Risk management)		X (Ciguatera)
Chinain 2020 [51]		X (database)	X (Risk management)	X (Marine toxin*)	X (Ciguatera)
Clausing 2016 [39]	X (website)	X (data)	X (Risk assessment)		X (Ciguatera)
Clua 2011 [40]		X (database)	X (Risk management)		X (Ciguatera)
Cressey 2019 [52]		X (database)	X (Risk assessment)	X (Marine toxin*)	X (Ciguatera)
FAO/WHO 2020 [53]		X (database)	X (Risk management)	X (Marine toxin*)	X (Ciguatera)
Friedman 2017 [43]	X (website)	X (database)	X (Risk assessment)	X (Marine toxin*)	X (Ciguatera)
Friedman 2019 [54]		X (database)	X (Risk assessment)	X (biotoxin*)	X (Ciguatera)
Goater 2011 [55]		X (database)	X (Risk assessment)		X (Ciguatera)
Beaty 2019 [35]	X (social media)	X (database)	X (Risk management)		X (Ciguatera)
Hallegraeff 2021 [66]		X (database)	X (Risk management)	X (biotoxin*)	X (Ciguatera)
Hian 2018 [50]		X (big data)	X (Risk assessment)		X (Ciguatera)
Kappel 2020 [56]		X (database)	X (Risk)	X (Marine toxin*)	X (Ciguatera)
Kibler 2017 [45]	X (website)	X (database)	X (Risk assessment)		X (Ciguatera)
Kusche 2021 [57]		X (database)	X (Risk management)	X (biotoxin*)	X (Ciguatera)
Leonardo 2021 [48]	X (smartphone)			X (Marine toxin*)	X (Ciguatera)
Li 2020 [34]	X (social media)	X (database)		X (Marine toxin*)	X (Ciguatera)
Liefer 2021 [68]		X (dataset)	X (Risk assessment)		X (Ciguatera)
Llewellyn 2010 [58]		X (database)	X (Risk)		X (Ciguatera)
Loeffler 2021 [33]	X (digital technologies)	X (database)	X (Risk management)	X (Marine toxin*)	X (Ciguatera)
Mustapa 2015 [37]	X (software)	X (database)	X (Risk)	X (biotoxin*)	X (Ciguatera)
Parsons 2012 [59]		X (database)	X (Risk assessment)	X (Marine toxin*)	X (Ciguatera)
Schoelinck 2014 [36]	X (software)	X (database)	X (Risk)		X (Ciguatera)
Solino 2020 [70]		X (data)	X (Risk assessment)	X (Marine toxin*)	X (Ciguatera)
Sunesen 2021 [60]		X (database)	X (Risk management)	X (Marine toxin*)	X (Ciguatera)
Tester 2010 [44]	X (website)	X (database)	X (Risk assessment)	X (Marine toxin*)	X (Ciguatera)
Tsagkaris 2019 [47]	X (smartphone)		X (Risk assessment)	X (Marine toxin*)	X (Ciguatoxin*)
Yang 2016 [61]		X (database)	X (Risk management)	X (biotoxin*)	X (Ciguatera)
Young 2020 [62]		X (database)	X (Risk)	X (Marine toxin*)	X (Ciguatera)
Zheng 2020 [67]		X (dataset)	X (Risk management)		X (Ciguatera)

Zingone 2021 [14]	X (database)	X (Risk)	X (Marine toxin*)	X (Ciguatera)

[1] refer to main manuscript for the reference list. [2] asterisk was used as a wildcard symbol to retrieve all possible variations of the relevant search term.

Table S2. Indicative social media accounts potentially relevant to CFP risk analysis.

Social Medium/ Account Title	Description	Link *
**Facebook**		
CiguaPIRE	International research team working to advance a global understanding on the origin of ciguatera	https://www.facebook.com/groups/205977216922912/
Ciguatera-Online	Provides a wide range of information on CFP, latest news about research improvement, information documents, links concerning CFP and the opportunity to share your experience and ask questions.	https://www.facebook.com/ciguateraonline
Ciguatera Fish Poisoning Awareness	Public service. This page serves to inform the public regarding Ciguatera Fish Poisoning, and ways to prevent it, specifically in Florida	https://www.facebook.com/CiguateraPoisoningFlorida/
Ciguatera fish poisoning - Project Walk For Health Alaska 2016	Create awareness of Ciguatera fish poisoning	https://www.facebook.com/CFPWalkForHealth
Ciguatera Support Group	Support Group for Sufferers of Ciguatera Poisoning	https://www.facebook.com/Ciguatera-Support-Group-178962905543570/
Ciguatera Poisoning	Ciguatera, How I Was Poisoned: Raising awareness on the disease	https://www.facebook.com/Ciguatera-Poisoning-129087550589584
Ciguatera Support and Information	Community for those interested in Ciguatera (suspect or suffering from the disease or simply interested)	https://www.facebook.com/groups/560312960832342
Fisheries NT - The Department of Industry, Tourism and Trade	Government Organization – Linked to Recreational fishing: Information and rules for fishing in the Northern Territory (Australia) - contains information relevant to ciguateric fish species	https://www.facebook.com/FisheriesNT
Fishing Central Queensland	Page all about fishing the Central Queensland region – contains information relevant to ciguateric fish species	https://www.facebook.com/groups/132451282855/
Florida's Poison Control Centers	Provides free, 24 hours a day, confidential information and treatment advice - contains information relevant to ciguatera poisoning (symptomatology/treatment)	https://www.facebook.com/FloridasPCC/
Greater Caribbean Center for Ciguatera Research	The GCCCR is a center of excellence in the study of ciguatera poisoning. The goal is to understand environmental impacts of toxin production, movement of toxins in the environment, and how human cells deal with toxin exposure	https://www.facebook.com/ciguacohh/
Hawaii Spearfishing & Freediving	Hawaii Spearfishing and Freediving group with conservation and safety emphasized - contains information relevant to ciguateric fish species	https://www.facebook.com/groups/HSFGroup/
Institute for Marine and Antarctic Studies - IMAS	Enhancing environmental understanding and facilitating thoughtful and sustainable development for the benefit of Australia and the world - contains information relevant to ciguateric fish species	https://www.facebook.com/InstituteforMarineandAntarcticStudies/
Instituto Tecnológico de Canarias - ITC.GobCan	Public service of the Canary islands Government, with activity in the fields of investigation, research and innovation - contains information relevant to ciguatera	https://www.facebook.com/ITC.Gobcan/
State of Hawaii Department of Health-Disease Outbreak Control Division	The mission is to protect and improve the health and environment for all people in Hawaii - contains information relevant to ciguatera poisoning (case reports/advice).	https://www.facebook.com/HI.DOCD/

**Social Medium/ Account Title**	**Description**	**Link ***
**Twitter (#ciguatera)**		
@alertoxnet	An Interreg Atlantic Area network for emerging Marine Toxins	https://twitter.com/alertoxnet
CiguaPIRE	International research team working to advance a global understanding on the origin of ciguatera	https://twitter.com/CiguaPire
Ciguatera (@CiguateraPR)	Spreading awareness of ciguatera fish poisoning.	https://twitter.com/ciguaterapr?lang=el
Codex Alimentarius	The official account of the FAO/WHO/Codex Alimentarius tweeting on International Standard setting and food safety issues	https://twitter.com/FAOWHOCodex
EFSA	European Food Safety Authority - Trusted science for safe food ǀ	https://twitter.com/EFSA_EU
FisheriesAquaculture	UN Food & Agriculture Organization's Fisheries & Aquaculture.	https://twitter.com/FAOfish
Got Food Poisoning? @iwaspoisoned	Web service for tracking reported food poisoning incidents	https://twitter.com/iwaspoisoned
IEO Vigo	Centro Oceanográfico de Vigo (Instituto Español de Oceanografía - IEO)	https://twitter.com/IEOVigo
NEPT NEGRIL	NGO responsible for protection and conservation of biodiversity of the Negril Environmental Protection Area	https://twitter.com/NEPT_NEGRIL
Seafood Safety team	Research led by Shauna Murray at @UTS_Science	https://twitter.com/SeafoodSafe_UTS

*All links accessed on 25 September 2021.
